# Surface-Functionalised Copper Oxide Nanoparticles: A Pathway to Multidrug-Resistant Pathogen Control in Medical Devices

**DOI:** 10.3390/nano14231899

**Published:** 2024-11-26

**Authors:** James Hall, Subbareddy Mekapothula, Rebecca Coxhill, Dominic Craske, Adam M. Varney, Gareth W. V. Cave, Samantha McLean

**Affiliations:** School of Science and Technology, Nottingham Trent University, Clifton Lane, Nottingham NG11 8NS, UK; jim.hall@ntu.ac.uk (J.H.); subba.mekapothula@ntu.ac.uk (S.M.); becky.coxhill@ntu.ac.uk (R.C.); dominic.eberl-craske@ntu.ac.uk (D.C.); adam.varney@ntu.ac.uk (A.M.V.)

**Keywords:** copper oxide nanoparticles, ESKAPE pathogens, 3-mercaptopropyltrimethoxysilane, MPTMS, antimicrobial activity, multi-drug resistance, glutamic acid, Glu–CuO nanoparticles, tissue culture, CDC reactor

## Abstract

Copper oxide nanoparticles (CuONPs) offer promising antimicrobial properties against a range of pathogens, addressing the urgent issue of antibiotic resistance. This study details the synthesis of glutamic acid-coated CuONPs (GA-CuONPs) and their functionalisation on medical-grade silicone tubing, using an oxysilane bonding agent. The resulting coating shows significant antimicrobial activity against both Gram-positive and Gram-negative bacteria, including multidrug-resistant strains, while remaining non-toxic to human cells and exhibiting stable adherence, without leaching. This versatile coating method can be applied during manufacturing, or for ad hoc modifications, enhancing the antimicrobial properties of medical devices.

## 1. Introduction

Antibiotics have been used for almost a century, since the discovery of the first true antibiotic, penicillin, by Sir Alexander Fleming, in 1928. This discovery revolutionised the treatment of bacterial infections and gave rise to the golden age of antibiotics in the 1950s and 1960s. However, the increased use and misuse of antibiotics has given rise to an inevitable increase in antibiotic resistance. This resistance was predicted by Sir Alexander Fleming as early as 1945, when he warned that the misuse and overuse of antibiotics could lead to resistance [1]. Today, we stand at the precipice of a post-antibiotic era, where the treatment of infections has become significantly more challenging and immunosuppressive therapies and conditions substantially riskier. In 2019 alone, it was reported that 1.27 million deaths globally were directly attributable to antibiotic-resistant infections [2], and it is conservatively estimated that if we do nothing to stem the tide of resistance, then by 2050 ten million people per annum will die from antibiotic-resistant infections, making antibiotic-resistant infections the single greatest cause of death globally [3]. It is, therefore, vital that alternatives to antibiotics are explored, with research into antimicrobials, both new and old. Ancient antimicrobials, including metals, have been used throughout history, as early as the Ancient Egyptian era, where silver and copper were used to treat burn wounds, which ultimately led to modern day metallic therapeutics and treatments [4]. Developing these ancient remedies in the 21st century, we have seen the emergence of nanoparticle technologies and the application of antimicrobial metals, particularly an increase in silver nanomaterial research, which is now commonly utilised for its antipathogenic properties, with applications ranging from food storage to wound dressings [5]. However, there are a growing number of reports on silver resistance amongst bacterial pathogens [6]. In addition, silver is susceptible to oxidation and a subsequent reduction in antipathogenic properties. It is also reported to have elevated cytotoxic effects in mammals, including reduced cell viability, lactate dehydrogenase leakage, and the generation of reactive oxygen species [7].

A viable and emerging alternative to silver, as an antimicrobial, is copper, which was utilised during the SARS-CoV-2 pandemic for its antiviral properties and was officially classified as an antimicrobial metal by the Environmental Protection Agency in 2008 [8,9,10]. Copper and its oxides are widely used as touch surfaces to reduce the spread and build-up of pathogens. Nanoparticles offer excellent surface area to volume ratios, as well as having excellent interaction rates with bacterial cells [11]. Therefore, the development of copper nanoparticles as antimicrobial agents harnesses the antimicrobial potential of copper, whilst utilising the advantageous properties of nanoparticle technologies, presenting a promising avenue of investigation. Further functionalisation of the nanoparticles’ surface with organic (amino acids) and inorganic ligands, which increases bioavailability and ingestion by pathogenic bacteria, is being explored [10,12].

One important application of antimicrobial research is in developing coatings for medical device materials. Advances in modern medicine mean that implanted medical devices are increasingly utilised to support and improve the patient’s quality of life. Several million implants are being placed globally each year, with the most prevalent thought to be intravascular devices, orthopaedic implants, dental implants, and cardiovascular devices [13]. Additionally, the number of patients requiring mechanical ventilation and, therefore, the placement of endotracheal tubes, is estimated to be up to 20 million per year [14]. This reliance on medical materials contacting the body provides an excellent scaffold for microbial contamination and subsequent infection in, often, vulnerable patients. Therefore, developing antimicrobial coatings for these devices will have a significant impact on the prevalence and severity of host infections.

Herein, we report on the application of an amino acid functionalized copper oxide nanoparticle as an antipathogenic coating, suitable for a range of medical device materials, including silicone, stainless steel, titanium, and polyvinyl chloride. Subsequently, both dip and spray coating techniques were utilised to apply the coating and antimicrobial activity was demonstrated against a range of clinically relevant bacterial pathogens.

## 2. Materials and Methods

### 2.1. Reagents and Instruments

All the chemicals and solvents were purchased from Merck, Gillingham, UK, unless otherwise stated, as reagent grade or LC–MS grade, and used without further purification. Copper (II) chloride anhydrous (Glentham Life Sciences, Corsham, UK) and sodium hydroxide (Glentham Life Sciences, Corsham, UK) were purchased. Dulbecco’s Modified Eagle medium, Dulbecco’s phosphate buffered saline, foetal bovine serum, dimethyl sulfoxide, and a penicillin–streptomycin solution, were purchased from Thermo Fisher Scientific, Loughborough, UK. Centers for Disease Control and Prevention (CDC) reactor coupons were purchased in stainless steel 316, titanium, polyvinyl chloride, and silicone, from BioSurface Technologies, Montana, MT, USA.

### 2.2. Synthesis of Copper Oxide Nanoparticles via Aqueous Precipitation

Copper oxide nanoparticles were synthesized via aqueous precipitation, following a modified procedure described in the literature [15]. Briefly, copper (II) chloride (100 mL, 0.1 M) and sodium hydroxide (100 mL, 0.2 M) aqueous solutions were pumped (60 mL/min) onto the centre of a rotating concave disc (10.5 cm diameter, 18 3 × 3 mm stepped ridges, 1000 rpm). The resultant solution was filtered and washed with deionized water three times, then placed in a vacuum rotary pump at 60 °C, until a fine powder formed, and excess water content was removed, which resulted in a yield of 6.42 g, 80.9%.

### 2.3. Glutamic Acid Coating of Copper Oxide Nanoparticles

The surface of the copper oxide nanoparticles was functionalized with glutamic acid via a mechanochemical extruder [15]. Briefly, copper oxide nanoparticles (25 g, 1 eq.) and glutamic acid (25 g, 1 eq.) were ground and stored in an airtight container under nitrogen until further use. Subsequently, glutamic acid-coated copper oxide nanoparticles were characterised by scanning electron microscopy (SEM), transmission electron microscopy (TEM), powder X-ray diffraction (XRD), dynamic light scattering (DLS), thermogravimetric analysis (TGA), and Fourier transform infrared spectroscopy (FTIR).

### 2.4. Characterisation of Glutamic Acid–Copper Oxide Nanoparticles

#### 2.4.1. Transmission Electron Microscopy

Transmission electron microscopy (TEM) was performed using a JEOL 2100 Transmission Electron Microscope (JEOL, Tokyo, Japan). The suspension of glutamic acid-coated copper oxide nanoparticles in water (10 µL) was loaded onto Holey carbon TEM grids (TAAB, Berkshire, UK), which were air dried at room temperature for 30 min. Excess suspension was removed prior to placing the Holey carbon grid into the TEM sample holder and the imaging taking place. Approximately 50 nanoparticles were analysed to measure the particle size and the distribution of the nanoparticle size, using ImageJ software (Version 1.53, NIH, Bethesda, MD, USA).

#### 2.4.2. Dynamic Light Scattering and Zeta Potential Measurements

Dynamic light scattering (DLS) and zeta-potential measurements were carried out using a Zetasizer nano series lab system (Malvern, UK), to measure the hydrodynamic size and the zeta potential of both the uncoated and glutamic acid–copper oxide nanoparticles. Nanoparticles in water at a certain concentration (1 mg mL^−1^) were loaded into a cuvette and measured for 100 repeats, at room temperature (20 °C). The zeta potential was measured by filling a folded capillary zeta cell (Malvern, UK) with copper oxide nanoparticles in water, at room temperature (20 °C), with a concentration of 1 mg mL^−1^.

#### 2.4.3. Thermogravimetric Analysis

Thermogravimetric analysis (TGA) was performed, using a TGA 4000 thermogravimetric analyser (PerkinElmer, Waltham, MA, USA), to analyse the weight of the glutamic acid coating on the copper oxide nanoparticles and on the glutamic acid–copper oxide nanoparticles on silicone material. Glutamic acid–copper oxide nanoparticles (50 mg) were added into a ceramic crucible at room temperature and placed into the TGA 4000 thermogravimetric analyser. The temperature of the TGA 4000 thermogravimetric analyser was increased in increments of 5 °C, every minute, until a temperature of 900 °C was achieved. This temperature was held for 30 min and, subsequently, brought back to room temperature.

### 2.5. Adhesion of Glutamic Acid–Copper Oxide Nanoparticles to Material Surfaces

The adhesion of glutamic acid–copper oxide nanoparticles to silicone tubing was achieved through a three-step dip-coating process, with each step involving a one-minute submersion, under gentle agitation. The tubing was first treated with a 0.1 M solution of 3-mercaptopropyltrimethoxysilane (MPTMS), followed by a 0.1 *w*/*w* glycerol mixture and, finally, a glutamic acid–copper oxide nanoparticle suspension (concentration stated for each experiment). The coated tubing was then UV sterilized for 30 min, before testing.

The CDC coupons (titanium, stainless steel, silicone, and PVC) were spray coated using three applications of MPTMS (0.1 M). Immediately afterwards, three sprays of glutamic acid–copper oxide nanoparticles (325.5 mg L^−1^) were applied. The compounds were prepared in a 50 mL spray bottle and sprayed from a distance of 30 cm, with 1.2 mL released per spray stroke. The coupons were UV cured using a wavelength of 254 nm in a UV box (Formlabs Foam Cure, Somerville, MA, USA) for 30 min, prior to the downstream application.

### 2.6. Leaching Studies on Glutamic Acid-Coated Copper Oxide Nanoparticles from Silicone Tubing

The leaching properties of copper from the nanoparticle-coated silicone tubing were determined according to the modified standard ISO 17294-2:2023 [16]. The leaching properties of copper from the silicone were investigated in solution via inductively coupled plasma mass spectrometry. A nanoparticle-coated silicone tube (10 cm length, 325 mg mL^−1^) was placed in line with a 300 series cased peristaltic pump (Watson-Marlow, Falmouth, UK), with a flow rate of 0.75 mL min^−1^, for 24 h, to allow 1.08 mL of artificial saliva medium to pass through the coated tube. After the medium passed through the tube, eluent (1 mL) was digested at a ratio of 1:10, in 70% nitric acid. The digested sample was further diluted in 2% nitric acid, until a one part per billion dilution was achieved. The spray-coated CDC coupons, in 24-well plates (Merck, Darmstadt, Germany), had 2 mL of plasma-like medium added to each well and were oscillated on a bench-top shaker bed, at 150 rpm, for 24 h. The coupons were removed and 1 mL of medium was taken for digestion and dilution, amounting to one part per billion (ppb). The ICP–MS elemental analysis was carried out using standard calibration (0–1000 ppb), using Certipur^®^ ICP Single-Element standards for copper and indium (20 ppb) as an internal standard.

### 2.7. Characterisation of Nanoparticle-Coated Materials

The glutamic acid–copper oxide nanoparticle-coated silicone tubing and CDC coupons were examined under a JEOL JSM-7100F Scanning Electron Microscope (SEM) (JEOL, Tokyo, Japan) and energy-dispersive X-ray spectroscopy (EDS) was performed using a X-Max^n^ EDS detector (Oxford Instruments, Oxford, UK). The samples were adhered using conductive copper tape to aluminium stubs (TAAB, UK) and placed into a sample holder. The PVC CDC coupon was coated with 10 nm of conductive carbon and the silicone tubing was coated with 5 nm of gold, both via a rotary pump sputter coater (Quorum Q150R ES, Lewes, UK), to increase the sample’s conductivity. The stainless-steel and titanium coupons were not coated. An accelerating voltage of 5 keV was used to take images of the coated coupons, using secondary electron imaging (SEI); an accelerating voltage of 5 keV was also used for the energy-dispersive X-ray spectroscopy analysis, to minimise the interaction volume.

### 2.8. Bacterial Strains, Mammalian Cell Lines, Growth Conditions, and Testing

The PS_Acine9 and PS_Acine7 strains were obtained from Nottingham Trent University and used with the permission of Professor Lesley Hoyles. The study of these anonymised isolates for use in non-commercial research, beyond the diagnostic requirement, was approved by an NHS research ethics committee (number 06/Q0406/20). *Staphylococcus aureus* USA 300 LEC2 and *Escherichia coli* O157:H7 were a kind gift from the Poole group at the University of Sheffield [17,18], UK. *Staphylococcus epidermidis* ATCC 12228 was obtained from the American Type Culture Collection. Clinical isolates of *Klebsiella pneumoniae* and *Pseudomonas aeruginosa* were purchased from Nottingham University Hospitals (NUH Trust) Pathogen Bank, under MTA, with permission granted for publication. Bacterial strains were grown in Mueller–Hinton growth medium, artificial saliva medium [19], or plasma-like medium [20,21], as appropriate. An immortalised human keratinocytes cell line (HaCaT) was obtained from Dr Elvina Chrysanthou, Nottingham Trent University, UK.

#### 2.8.1. Minimum Inhibition and Bactericidal Concentration Assays

Minimum inhibitory concentration (MIC) assays were performed as previously described [22]. The assays were performed with glutamic acid–copper oxide nanoparticles in biological triplicate, for each strain tested. The plates were incubated for 18 h at 37 °C. The lowest concentration of Glu–CuONPs that showed no visible growth was designated as the minimum inhibitory concentration. Following the MIC assay, 10 μL from each well displaying no visible growth was transferred onto an MHA plate, along with a positive growth control. The plates were incubated for 18 h at 37 °C. The lowest concentration showing no growth was recorded as the minimum bactericidal concentration (MBC).

#### 2.8.2. Toxicity Assays for Glutamic Acid–Copper Oxide Nanoparticles and 3-Mercaptopropyltrimethoxysilane

Colorimetric MTT (3-(4,5-Dimethylthiazol-2-yl)-2,5-diphenyltetrazolium bromide) assays were used to measure the toxicity of glutamic acid–copper oxide nanoparticles and 3-mercaptopropyltrimethoxysilane (MPTMS) in regard to a human epidermal keratinocyte cell line. HaCaT cells were split until the seventh passage was achieved and all the experiments were performed on this passage. The cells were grown in Dulbecco’s Modified Eagle medium to confluency and diluted in fresh medium to a concentration of 2 × 10^5^ cells mL^−1^. The diluted solution (100 µL) was added to each well and incubated for 24 h at 37 °C, allowing the cells to adhere to the well. The medium was replaced with fresh medium containing glutamic acid–copper oxide nanoparticles, at concentrations ranging from 500 µg mL^−1^ to 275 µg mL^−1^. For MPTMS, ISO 10993-5:2009 was utilised at concentrations ranging from 0.05 to 1 M [23]. Briefly, the silicone-tubing surface (3 mm × 3 mm) was coated with MPTMS and cured. These surfaces were added to confluent HaCaT cells with an additional control well, with sterile untreated silicone and MTT assays performed after contact.

#### 2.8.3. Modified Minimum Biofilm Eradication Concentration Assay

The modified minimum biofilm eradication concentration (mMBEC) assay was performed using 24-well plates, with 1 cm of silicone tube cuttings adhered to the lid of the plates, such that each piece of tubing was suspended into each of the 24-wells when the lid was closed, without contacting the well. These tube cuttings were subsequently dip coated, as described in Section 2.5, and tested against each bacterial species to determine the change in the biofilm biomass formation and in viable cells recovered from the material. Each well was filled with Mueller–Hinton broth (1800 µL) and inoculated with each species (200 µL) in Mueller–Hinton broth and diluted to a concentration of 10^6^ Colony Forming units per mL (CFU mL^−1^). The biofilm biomass was measured by submerging the silicone tubes in crystal violet (0.1%) for two hours, gently washing with sterile phosphate buffered saline, and submerging the detached silicone tubes in ethanol to recover the crystal violet. Optical density measurements were taken at 550 nm. Viable cell counts were carried out by washing the silicone tubes with sterile phosphate buffered saline and resuspending the biofilms in sterile phosphate buffered saline (2 mL) by vortexing for 1 min rounds, until all the biomass was removed and aggregates resuspended. The recovered cells were diluted in phosphate buffered saline and plated onto Mueller–Hinton agar to count the viable cells recovered.

#### 2.8.4. Centers for Disease Control and Prevention (CDC) Bioreactor Assay

The CDC bioreactor coupons, spray coated with glutamic acid–copper oxide nanoparticles, were placed into a sterile CDC bioreactor, containing a plasma-like medium (500 mL). The bioreactor was inoculated with *Staphylococcus aureus* (1 mL) at 10^8^ CFU mL^−1^ and incubated at 21 °C for 24 h, at 125 rpm, after which the CDC coupons were removed aseptically and assessed for biofilm biomass and viable cell measurements, as described in Section 2.8.3.

## 3. Results and Discussion

### 3.1. Characterisation of Copper Oxide Nanoparticles and Glutamic Acid–Copper Oxide Nanoparticles

The synthesis of copper oxide nanoparticles is well-studied using various synthesis methods and, particularly, co-precipitation allows excellent control of the size and shape of the nanoparticle, which can be achieved through controlling the temperature, reaction time, and mixing rate, via a spinning disc reactor or magnetic flea [24,25]. By altering the flow rates of precursor solutions and the rate of rotation of the spinning disc reactor or magnetic flea, the size of the nanoparticle can be increased or decreased, with increasing temperatures correlating with an increase in the nanoparticle size and slower rotational speeds correlating with an increase in the nanoparticle size.

**Figure 1:**
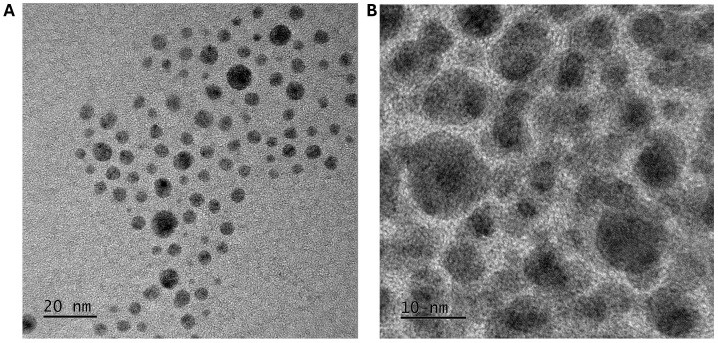
Transmission electron micrograph of copper oxide nanoparticles. (**A**) Copper oxide nanoparticles synthesised by aqueous precipitation show a spherical shape, with a size of 5.7 ± 3.5 nm. (**B**) Glutamic acid–copper oxide nanoparticles, with a size measuring approximately 30.6 ± 6.7 nm, show a darker nanoparticle core, surrounded by the organic glutamic acid material.

Copper oxide nanoparticles were synthesised via aqueous co-precipitation and their shape and morphology characterised using TEM (Figure 1A). ImageJ analysis showed that the size of the synthesised nanoparticles was 5.7 ± 3.5 nm (Figure S1). The hydrodynamic size, polydispersity, and zeta potential were also measured to determine the nanoparticle’s stability in an aqueous solution. The hydrodynamic size of the nanoparticles was measured to be 74.93 ± 5.170 nm, with a polydispersity index of 0.35 (Figure S2).

The zeta potential of these nanoparticles was also measured, showing a potential of −0.4 ± 6.2 mV, indicating that these nanoparticles would not be stable in an aqueous solution, as the required values for particles to be stable are typically ±30 mV [15]. As the downstream application required water solubility, and in order to increase the bioavailability of the copper oxide nanoparticles, the nanoparticles were coated in glutamic acid via non-covalent electrostatic interactions and their characteristics were compared. The TEM analysis, showing spherical-shaped nanoparticles, showed significant organic ligand content on the nanoparticle surface (Figure 1B). ImageJ analysis showed an increase in the nanoparticle size to 30.6 ± 6.7 nm (Figure S3). The dynamic light scattering measurements showed that the nanoparticles had a polydispersity index of 0.06 and the hydrodynamic size had increased to 123.1 ± 12.7 nm (Figure S4). The zeta potential of the glutamic acid–copper oxide nanoparticles was measured as −42.0 ± 3.2 mV, indicating that the particles would remain stable in aqueous solutions. Thermogravimetric analysis measured the weight ratio between the glutamic acid and the copper oxide nanoparticle at 1:1 (*w*/*w*) (Figure S5). The nanoparticles could not be tested against the HaCaT cells without a coating due to precipitation.

### 3.2. Antimicrobial Evaluation of the Glutamic Acid–Copper Oxide Nanoparticles

To understand whether the synthesised Glu–CuO nanoparticles were antimicrobial, they were tested against a panel of clinically isolated pathogens, using minimum inhibitory (MIC) and minimum bactericidal concentration (MBC) assays. The panel of bacteria pathogens were first assessed for their resistance to common antibiotics. A range of clinical sensitivities and resistances were observed across the panel, with the two *Acinetobacter* species being classified as multidrug resistant [26] (Table S1). The growth of all the bacterial pathogens within this panel was inhibited by the glutamic acid–copper oxide nanoparticles and all the strains were killed at slightly higher concentrations (Table 1). Importantly, there was no discernible difference in the antimicrobial activity between Gram-positive and Gram-negative pathogens, which have distinct cell envelopes that often show altered resistances to antibiotics. Neither was there a difference in the antimicrobial activity of the nanoparticles in regard to relatively antibiotic-sensitive strains versus resistant strains, indicating that their existing antibiotic-resistance mechanisms (against carbapenems, aminoglycosides, fluroquinolones, cephalosporins, and monobactams) do not confer resistance to glutamic acid–copper oxide nanoparticles.

### 3.3. Adhesion of Glutamic Acid–Copper Oxide Nanoparticles to Medical-Grade Materials Results in an Evenly Distributed Antimicrobial Coating

To further explore the antimicrobial activity and application of glutamic acid–copper oxide nanoparticles and their subsequent application as an antimicrobial coating for medically relevant materials, the nanoparticles were adhered to stainless steel, titanium, polyvinyl chloride (PVC), and silicone coupons, as well as silicone tubing, which is representative of commonly used medical grade tubing used within healthcare. The nanoparticles were bonded to the materials, after the application of MPTMS as the bonding agent. The silicone tubing was dip coated, whilst the medical device material coupons were spray coated.

**Figure 2:**
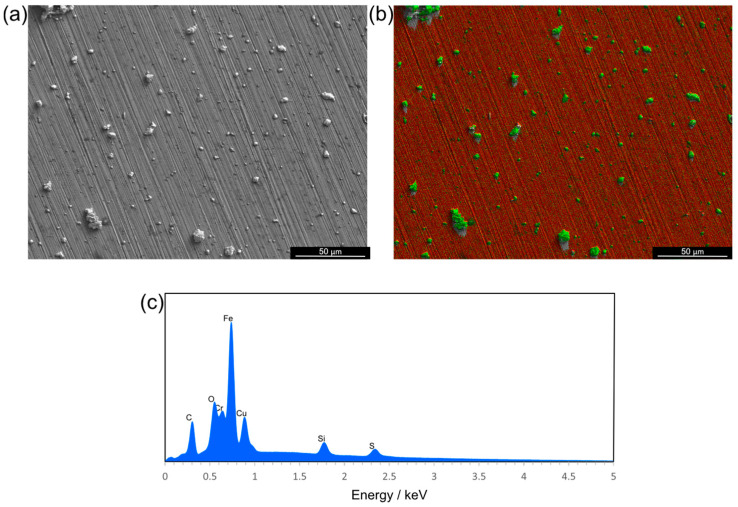
(**a**) Scanning electron micrograph (SEI) of stainless-steel CDC coupon surface after coating with Cu nanoparticles. (**b**) EDS mapping of same area as (**a**), red areas represent iron signal, green represents copper signal. (**c**) EDS map sum spectrum of (**b**). EDS and images taken at 5 kV accelerating voltage.

Scanning electron microscopy with energy-dispersive X-ray spectroscopy was performed to evaluate the coverage of the coating on the material surfaces (Figure 2 and Figure S6). Further quantification of the loading on the materials was performed using thermogravimetric analysis, finding that when using dip-coating and spray-coating techniques, the total copper loaded onto the material was 0.62% of the total mass of the tubing (Figure S7).

When developing antimicrobial coatings for medical devices, an important determination is whether the coating leaches into the environment over time, as this may have implications for the host. Therefore, leaching studies tested whether the copper separated from the silicone tubing into artificial saliva medium that was pushed through the tubing at a rate of 0.75 mL min^−1^ for a given total volume (1 L). An artificial saliva medium was chosen for this assay as a medium that is more representative of real-world infection than commonly used rich laboratory media, such as Mueller–Hinton broth. The research shows that bacterial pathogens behave differently in laboratory media compared with more physiologically relevant media; therefore, studies performed using laboratory media may not accurately reflect bacterial behaviours and sensitivity to antimicrobials in the environment of a real-world infection [27]. The artificial saliva medium that flowed through the tubing was analysed using inductively coupled plasma mass spectrometry to measure copper leaching from the coating, simulating conditions similar to those during mechanical ventilation. Under these conditions, copper leached from the tubing at a concentration of 8 ± 35 mg L^−1^ (Table S2), indicating minimal removal of the coating from the silicone tubing. There was agglomeration of the Glu–CuO nanoparticles on the surface of the material caused by the MPTMS during the curing stage; however, EDS analysis showed that copper oxide nanoparticles were still present across the surface of the material.

### 3.4. Human Cell Line Toxicity Assessment of Antimicrobial-Coating Components Identifies Concentrations That Can Be Safely Applied to Medical Materials

**Figure 3:**
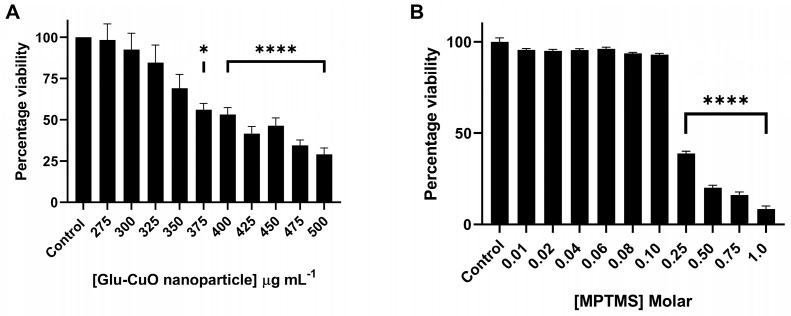
Cytotoxicity of human epidermal keratinocyte cells only occurs at high concentrations of glutamic acid–copper oxide nanoparticles and MPTMS. (**A**) Toxicity of glutamic acid–copper oxide nanoparticles in regard to HaCaT cells was measured via an MTT assay. (**B**) Toxicity of MPTMS was measured following ISO 10993-5:2009 [23]. Notes: *n* = 9 ± SEM, significance determined by one-way ANOVA test; * *p* > 0.05, **** *p* > 0.001, compared to no reagent present.

When developing an antimicrobial coating for materials that contact areas of the human body, it is essential to ensure that the coating remains non-toxic to the host. To understand whether the developed antimicrobial coating, described herein, was appropriate for contact with human cells, a viability assay was performed on a human epidermal keratinocyte (HaCat) cell line, after exposure to either glutamic acid–copper oxide nanoparticles or the bonding agent, MPTMS. For the nanoparticles, significant toxicity was only observed after 24 h of exposure to 375 µg mL^−1^ in solution (Figure 3A), a concentration almost 50-fold higher than the concentration of copper that leached from the tubing (~8 mg L^−1^). Oxysilanes, such as MPTMS, are known to be excellent adhesives in terms of oxides to other substrates, such as silicon wafers, metals, and PDMS [16], and were, therefore, a reasonable choice of bonding agent for the adhesion of the nanoparticles to medical device materials. However, the toxicity of MPTMS must also be accounted for when assessing the potential host toxicity of the coating. The toxicity of MPTMS to the HaCaT cell line was measured to ensure that toxicity was not observed at the working concentrations used for this binding agent. As MPTMS would only ever contact human cells after curing, the evaluation of its toxicity was also performed after the MPTMS had been cured, following ISO 10993-5:2009 [23]. In this assay, significant toxicity was observed only at concentrations greater than 0.25 Molar (Figure 3B). As the starting concentration used in the coating process was 0.1 M, this indicated that the method used was within the safe limits for exposure to human cells.

### 3.5. Antimicrobial Activity of Glutamic Acid–Copper Oxide Nanoparticle Coatings

**Figure 4:**
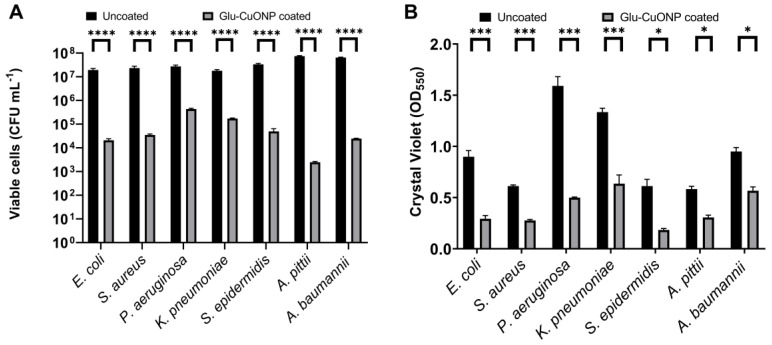
A reduction in the biofilm biomass accumulation and cell viability was observed for all bacterial species after exposure to glutamic acid–copper oxide nanoparticle-coated silicone tubing. Bacterial cultures were inoculated in 19 wells of a 24-well plate for each bacterial species tested and biofilm was allowed to form on the tubing, either coated with 20 times the minimum bactericidal concentration of glutamic acid–copper oxide nanoparticles (grey bars) or uncoated (black bars), for 24 h at 37 °C in artificial saliva medium. After incubation, three biological replicates (each performed in technical triplicate) were (**A**) diluted and cultured to determine the viable cells recovered and (**B**) stained with crystal violet and the resultant turbidity measured at OD_550_ to determine the total biofilm biomass. Notes: *n* = 9 ± SEM, statistical significance measured using unpaired *t* test; * *p* > 0.05, *** *p* > 0.005, **** *p* > 0.001.

To determine the antimicrobial activity of the glutamic acid–copper oxide nanoparticle coating a modification to the standard minimum biofilm eradication concentration (MBEC) assay was used. Here, instead of coating plastic pegs, 1 cm lengths of silicone tubing were adhered to the lid of sterile 24-well plates, so that they could be immersed in a solution and placed in the corresponding wells without touching the bottom or sides of the plate. This allowed the evaluation of the biofilm formation on the inner and outer surface of the tubing, which is a more accurate representation of real-world biofilm formation on medical tubing, such as endotracheal tubes. This modified MBEC (mMBEC) assay was used to evaluate the effect of the antimicrobial coating on both the total biofilm biomass accumulation and on the number of viable bacterial cells. Here, an artificial saliva medium was also used to mimic physiologically relevant conditions. We observed that at the minimal bactericidal concentration of glutamic acid–copper oxide nanoparticles for each bacterial species (identified in Table 1), a reduction in both the total biofilm biomass and number of viable cells was observed for all the bacterial pathogens; however, the extent of the reduction varied across the species tested (Figure S7). At 20 times the minimum bactericidal concentration, a significant reduction was observed for all the tested bacterial pathogens in regard to both the total biofilm biomass and cell viability (Figure 4).

### 3.6. The Glutamic Acid–Copper Oxide Nanoparticle Coating Remains Active Across a Range of Medical Device Materials

**Figure 5:**
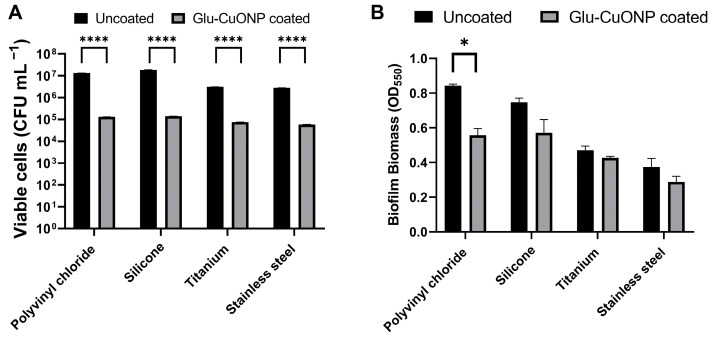
A reduction in biofilm biomass accumulation and cell viability was observed for *S. aureus* (MRSA) after exposure to numerous glutamic acid–copper oxide nanoparticle-coated medical materials. Coupons made of polyvinyl chloride, silicone, titanium, and stainless steel were spray coated with 20 times the minimum bactericidal concentration of glutamic acid–copper oxide nanoparticles (grey bars) or not coated (black bars) and placed in a CDC reactor. The reactors were inoculated with *S. aureus* and incubated for 24 h at 37 °C in a plasma-like medium. After incubation, the coupons removed and either (**A**) stained with crystal violet and the resultant turbidity measured at OD_550_ to determine the total biofilm biomass or (**B**) diluted and cultured to determine the number of viable cells recovered. Notes: *n* = 9 ± SEM, statistical significance measured using unpaired *t* test; * *p* > 0.05, **** *p* > 0.001.

To understand whether this antimicrobial coating could be applied across a range of medical device materials, including stainless steel, titanium, polyvinyl chloride, and silicone, CDC coupons were spray coated with glutamic acid–copper oxide nanoparticles and their activity was tested against a methicillin-resistant strain of *S. aureus* (MRSA), which is a pathogen of significance due to its impact on public health, its resistance to multiple antibiotics, and its prevalence in healthcare settings, leading to device contamination and subsequent infection risk. The assays were conducted in a CDC bioreactor, at 20 times the bactericidal concentration for this strain, in a plasma-like medium. This medium mimics the components found in human plasma and interstitial fluid, which provide a better indication of real-world antimicrobial activity than rich laboratory media and is used in cancer models to improve metabolic fidelity, for the same reason [21]. A plasma-like medium also reflects the variety of environments these medical device materials may be implanted into, including joint replacements, drainage tubes, and cardiovascular devices [13]. After incubation with the bacterial pathogen, the coated coupons were recovered and evaluated for both the total biofilm biomass and number of viable cells (Figure 5). A varied reduction in the total biofilm biomass was observed across the material types tested; however, a significant reduction in the number of viable cells was found for all the tested materials, suggesting that this antimicrobial is suitable for a variety of implant types.

The antimicrobial activity of glutamic acid–copper oxide nanoparticles observed against a range of bacterial pathogens, including both Gram-positive and Gram-negative species, and strains with a range of antimicrobial activities, highlights the multifaceted mechanism of action of copper as an antimicrobial. The primary mechanism of antimicrobial activity is thought to be via the generation of reactive oxygen species that interfere with bacterial cellular membranes [28,29]. However, this is unlikely to be the complete picture as reactive oxygen species are capable of DNA, protein, and lipid damage, as well as disrupting essential cellular processes. Resistance to copper antimicrobials, as with all antimicrobial therapies, remains a threat. Copper resistance has been observed in copper-polluted agricultural soils [30]; it is, therefore, important to reserve the use of copper antimicrobials for situations where the likelihood of infection is high and the consequences of infection are severe, such as to prevent the infection of vulnerable patients undergoing mechanical ventilation or other medical procedures.

## 4. Conclusions

Here, we show that copper oxide nanoparticles can be functionalised with glutamic acid to improve the stability of the particle in solution, whilst retaining their antimicrobial properties. These glutamic acid–copper oxide nanoparticles were successfully adhered to a range of medical-grade materials via both dip and spray coating, using MPTMS as an adhesive, including silicone, stainless steel, titanium, and polyvinyl chloride. The glutamic acid–copper oxide nanoparticles showed toxicity to human cells at concentrations of ≥375 mg L^−1^, far in excess of the 8 mg L^−1^ that leaches into media over 24 h, offering a significant therapeutic window for this antipathogenic coating. The agglomeration of Glu–CuO nanoparticles does not detrimentally affect the efficacy of the biocompatibility of the material, as the leached material concentration is far below the environmental toxicity limits. A range of antibacterial assays demonstrated the efficacy of this coating and its potential to reduce the incidence and severity of infections for patients undergoing mechanical ventilation or medical device implantation.

## Figures and Tables

**Table 1:** The minimum inhibitory and bactericidal concentrations of glutamic acid–copper oxide nanoparticles show antimicrobial activity against a range of Gram-positive and Gram-negative bacterial species. Note: *n* = 3 biological replicates (each performed in technical triplicate).

Bacterial Pathogen	Details/Origin	MIC (mg L^−1^)	MBC (mg L^−1^)
*Escherichia coli*	0157:H7 Poole RK, University of Sheffield	258	325
*Staphylococcus aureus*	USA 300 LAC JE2 Poole RK, University of Sheffield	258	325
*Pseudomonas aeruginosa*	Clinical isolate, neonatal sepsis Forsythe SJ, Nottingham Trent University	325	325
*Klebsiella pneumoniae*	Clinical isolate, neonatal enterocolitis Forsythe SJ, Nottingham Trent University	325	325
*Staphylococcus epidermidis*	ATCC 12228	258	325
*Acinetobacter pittii*	PS_Acine7: clinical isolate, bronchial lavage Hoyles L, Nottingham Trent University	258	325
*Acinetobacter baumannii*	PS_Acine9: clinical isolate, wound Hoyles L, Nottingham Trent University	258	325

## Data Availability

Data are available from libinfodirect@ntu.ac.uk.

## Supplementary Materials

The following supporting information can be downloaded at: https://www.mdpi.com/article/10.3390/nano14231899/s1, Figure S1: The measured size of copper oxide nanoparticles using transmission electron microscopy and ImageJ analysis. Figure S2: The hydrodynamic size of copper oxide nanoparticles measured by dynamic light scattering. Figure S3: The measured size of glutamic acid coated copper oxide nanoparticles using transmission electron microscopy and ImageJ analysis. Figure S4: The hydrodynamic size of glutamic acid coated copper oxide nanoparticles measure by dynamic light scattering. Figure S5: Thermogravimetric analysis of glutamic acid coated copper oxide nanoparticles determined the ratio of nanoparticle to amino acid. Figure S6: (a) Scanning electron micrograph of polyvinyl chloride CDC coupon surface after coating with Cu nanoparticles. (b) EDS mapping of same area as (a), red areas represent carbon signal, green represents copper signal. (c) EDS map sum spectrum of (b). EDS and images taken at 5 kV accelerating voltage. Figure S7: (a) Scanning electron micrograph of titanium CDC coupon surface after coating with Cu nanoparticles. (b) EDS mapping of same area as (a), red areas represent titanium signal, green represents copper signal. (c) EDS map sum spectrum of (b). EDS and images taken at 5 kV accelerating voltage. Figure S8: (a) Scanning electron micrograph of silicone CDC coupon surface after coating with Cu nanoparticles. (b) EDS mapping of same area as (a), red areas represent silicon signal, green represents copper signal. (c) EDS map sum spectrum of (b). EDS and images taken at 5 kV accelerating voltage. Figure S9: Copper oxide nanoparticle coating on silicone tubing showing the percentage mass difference between uncoated and coated with copper oxide nanoparticles. Table S1: Antibiotic disk diffusion data following the EUCAST guidelines and interpretation using EUCAST breakpoints (2022). Table S2: Leaching data taken from artificial saliva medium and water using ICP-MS. Copper present in the salts of the medium and water were subtracted from the leached media to assess the trace copper leached from the coated tubing. ICP-MS was repeated in biological triplicate and averaged across ten measurements. Error calculated by averages of thirty total measurements and combining artificial saliva medium plus the leached medium.
