# Supplementary material for: Surface-Functionalised Copper Oxide Nanoparticles: A Pathway to Multidrug-Resistant Pathogen Control in Medical Devices
Source: Nanomaterials (Basel). 2024 Nov 26;14(23):1899. doi: 10.3390/nano14231899 (PMC11643601; doi:10.3390/nano14231899)
Supplement: Supplementary file 1 [file nanomaterials-14-01899-s001.zip › nanomaterials-3280506-supplementary.pdf]

Electronic Supplementary Information for:

Surface functionalised copper oxide nanoparticles: A pathway to multidrug-resistant pathogen control on medical devices

James Hall [1], Subbareddy Mekapothula [1], Rebecca Coxhill [2], Dominic Craske [1], Adam M. Varney [2], Gareth W. V. Cave [1], and Samantha McLean [1,*]

[1]School of Science and Technology, Nottingham Trent University, Clifton Lane, Nottingham NG11 8NS, UK

[2]Medical Technologies Innovation Facility, Clifton Lane, Nottingham NG11 8NS, UK

*Correspondence: Gareth.Cave@ntu.ac.uk.uk, Tel.: +44 115 848 3242; Samantha.McLean@ntu.ac.uk; Tel.: +44 115 848 3324

S1. Materials and methods

Copper (II) chloride, sodium hydroxide and glutamic acid were purchased from Merck (United Kingdom, SP8 4XT). All chemicals and solvents were purchased as reagent grade, ICP-MS grade and used without further purification. Inductive Coupled Plasma-Mass Spectrometry (ICP-MS, Perkins Elmer Nexion1000) was used for elemental analysis of copper oxide nanoparticles. Dynamic light scattering (DLS, Zetasizer nano series) Scanning Electron Microscopy (SEM, JEOL JSM-7100F) and Transmission electron microscopy (TEM JEOL JEM-2100Plus) were used to analyse the structural morphology of copper oxide nanoparticles.

Strain PS_Acine9 and PS_Acine7 were used with the permission of Prof Lesley Hoyles, Nottingham Trent University. The study of this anonymized isolate for use in non-commercial research beyond the diagnostic requirement was approved by an NHS research ethics committee (number 06/Q0406/20). *Staphylococcus aureus* USA 300 LEC2 and *Escherichia coli* O157:H7 we a kind gift from the Poole group at the University of Sheffield, UK. *Staphylococcus epidermidis* ATCC 12228 was obtained from the American Type Culture Collection. All other species were acquired from Nottingham University Hospitals (NUH) Trust Pathogen Bank, under MTA with permission granted for publication.

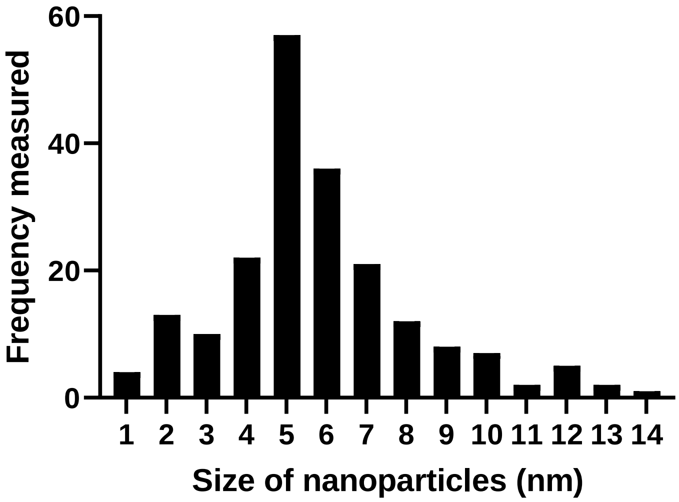

Figure S1 *The measured size of copper oxide nanoparticles using transmission electron microscopy and ImageJ analysis.*

Copper oxide nanoparticles were measured using ImageJ at 5.7 ± 2.3 nm according to measurement of 200 individual nanoparticles.

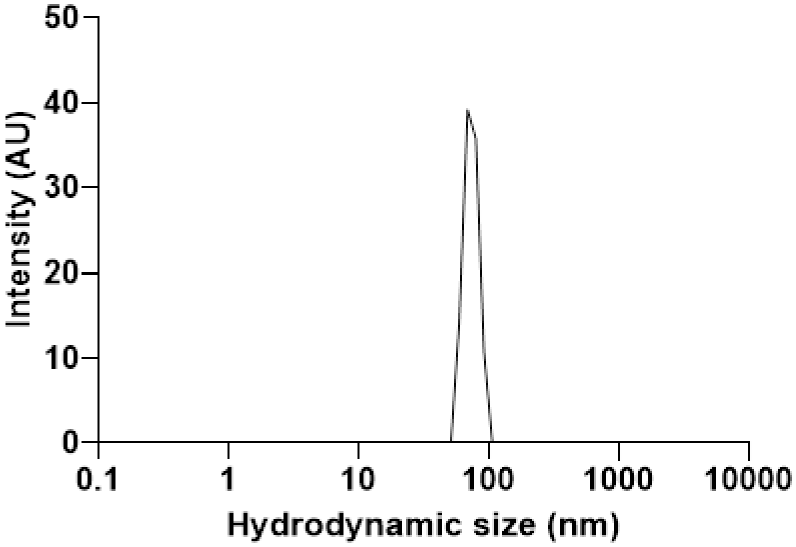

Figure S2 The h*ydrodynamic size of copper oxide nanoparticles measured by dynamic light scattering*

The hydrodynamic size of copper oxide nanoparticles was measured using a Zetasizer nano series at 74.93 ± 5.170 nm with a polydispersity index of 0.35.

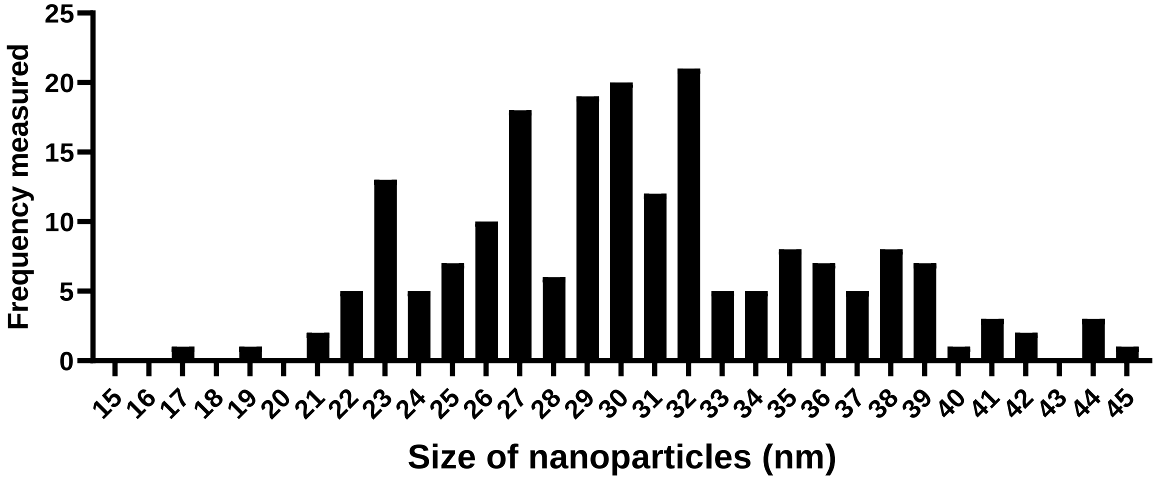

Figure S3 *The measured size of glutamic acid coated copper oxide nanoparticles using transmission electron microscopy and ImageJ analysis.*

Glutamic acid coated copper oxide nanoparticles were measured using ImageJ analysis at 30.6 ± 5.3 nm according to measurement of 200 individual nanoparticles.

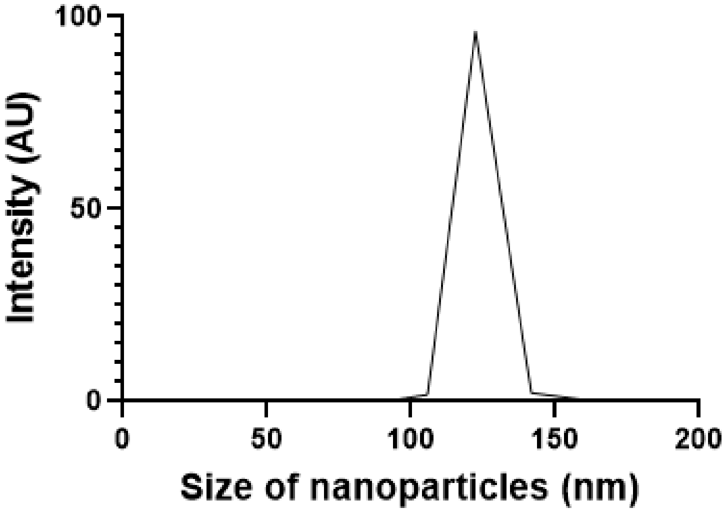

Figure S4 *The hydrodynamic size of glutamic acid coated copper oxide nanoparticles measure by dynamic light scattering*

The hydrodynamic size of glutamic acid coated copper oxide nanoparticles was measured using a Zetasizer nano series at 123.1 ± 12.77 nm with a polydispersity index of 0.06.

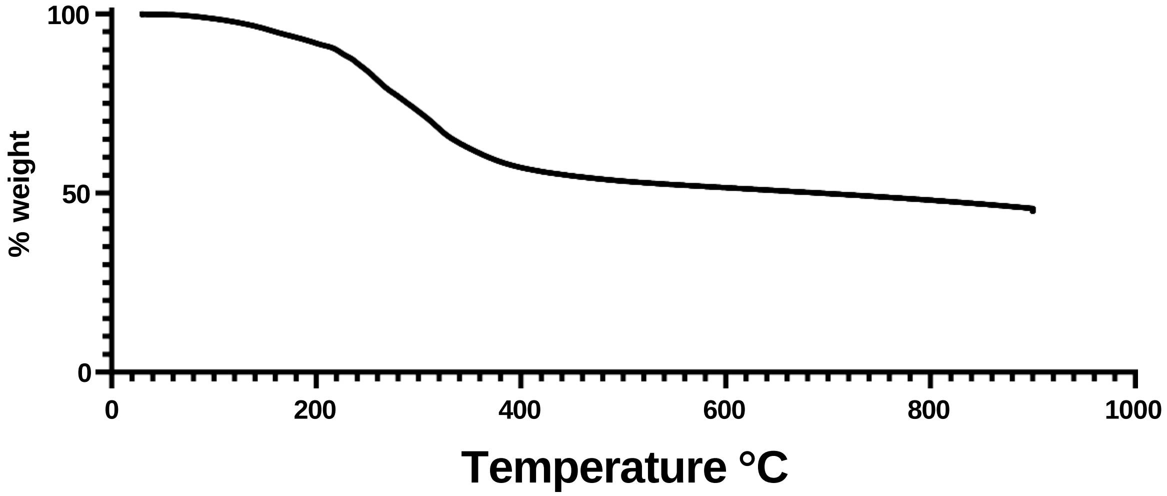

Figure S5 Thermogravimetric analysis of glutamic acid coated copper oxide nanoparticles determined the ratio of nanoparticle to amino acid.

The weight of copper oxide nanoparticles to glutamic acid coating was measured at 1:1.19 w/w by thermogravimetric analysis. N = 3 (the error is absorbed by the symbol size).

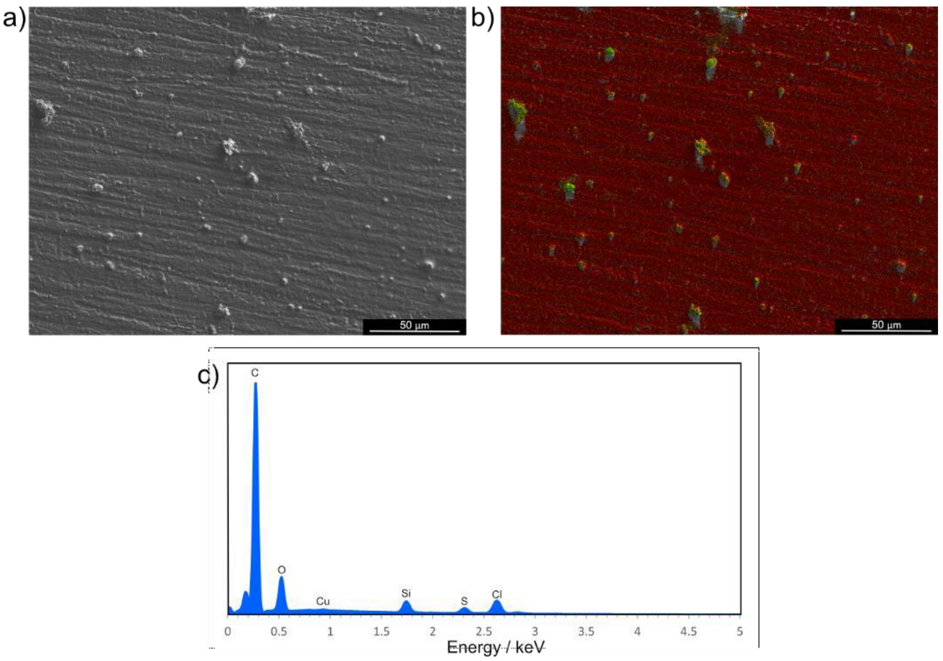

Figure S6 a) Scanning electron micrograph of polyvinyl chloride CDC coupon surface after coating with Cu nanoparticles. b) EDS mapping of same area as (a), red areas represent carbon signal, green represents copper signal. c) EDS map sum spectrum of (b). EDS and images taken at 5 kV accelerating voltage.

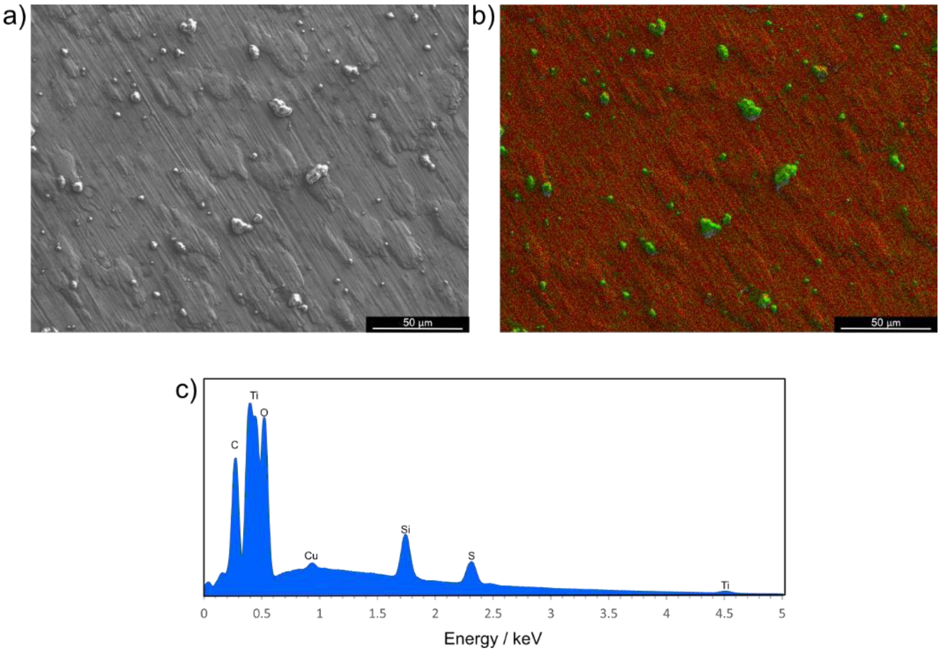

Figure S7 a) Scanning electron micrograph of titanium CDC coupon surface after coating with Cu nanoparticles. b) EDS mapping of same area as (a), red areas represent titanium signal, green represents copper signal. c) EDS map sum spectrum of (b). EDS and images taken at 5 kV accelerating voltage.

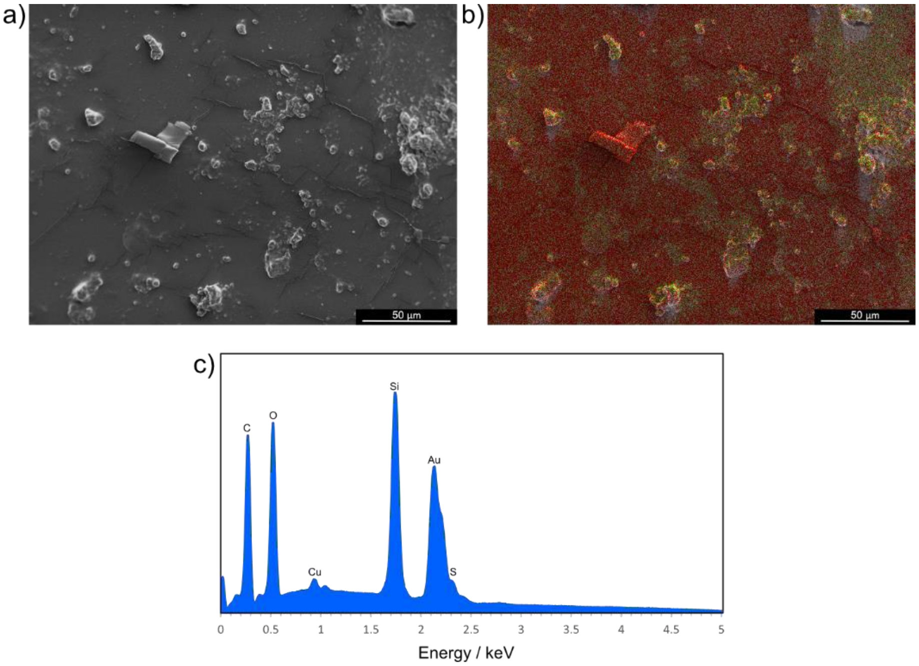

Figure S8 a) Scanning electron micrograph of silicone CDC coupon surface after coating with Cu nanoparticles. b) EDS mapping of same area as (a), red areas represent silicon signal, green represents copper signal. c) EDS map sum spectrum of (b). EDS and images taken at 5 kV accelerating voltage.

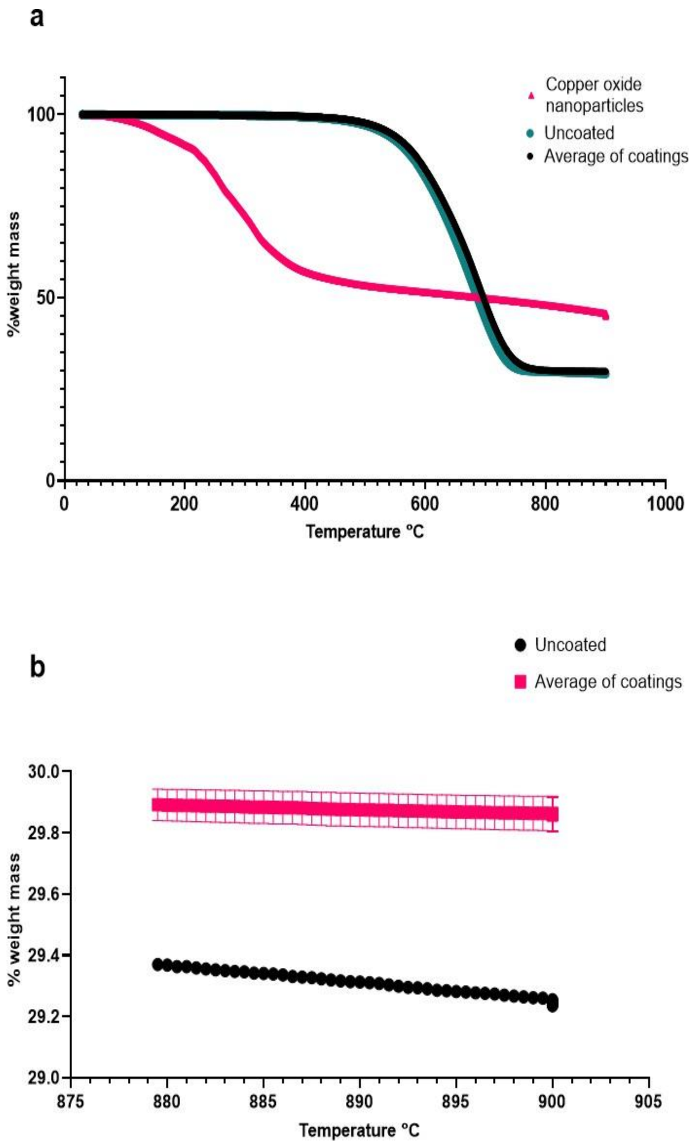

Fig. S9 Copper oxide nanoparticle coating on silicone tubing showing the percentage mass difference between uncoated and coated with copper oxide nanoparticles. a) The data for the full experiment where the difference between coated and uncoated silicone tubing is measured, including coated copper oxide nanoparticles showing the weight between copper oxide nanoparticles and the glutamic acid coating. Error given as standard error of mean, N = 3. Analysis showed that the final ratio of water, glutamic acid and copper oxide nanoparticles was determined (Fig. S6a: 1.34 ± 0.02%, 53.60 ± 0.02%, 45.06 ± 0.02% respectively. b) The average of the final 100 data points showing the difference between.). The data compared the coated

and uncoated mass of silicone tubing with final measurements indicating a total weight of 0.62 ± 0.007% difference (Fig. S6b).

Class	Strain	*Escherichia coli*	Clinical isolate *klebsiella pneumonia*	*Pseudomonas aeruginosa*	Clinical isolate *Acinetobacter pittii*	Clinical isolate *Acinetobacter baumannii*	*Staphylococcus aureus*	ATC 12228 *Staphylococcus epidermidis*	Interpretation of zone of inhibition compared to EUCAST 2022 breakpoint value
	Antibiotic	Zone diameter (mm)	Zone diameter (mm)	Zone diameter (mm)	Zone diameter (mm) *(mg ml^{-1})	Zone diameter (mm) *(mg ml^{-1})	Zone diameter (mm)	Zone diameter (mm)	Interpretation
Carbapenems	Doripenem	26.7	22	13.7	0	0	N/A	N/A	Susceptible
	Ertapenem	N/A	N/A	N/A	0	0	N/A	N/A	Resistant
	Meropenem	N/A	N/A	N/A	0	0	N/A	N/A	Susceptible, increased exposure
	Imipenem	N/A	N/A	N/A	0	0	N/A	N/A	
Aminoglycosides	Tobramycin	23.5	17.8	21.3	0	0	18	29.5	
	Amikacin	25.5	19.5	21.7	0	0	16.2	26.1	
	Gentamycin	24.6	18.7	N/A	0	0	18.7	30.2	
Fluoroquinolones	Levofloxacin	37.7	30.1	25.1	N/A	N/A			
	Ciprofloxacin	N/A	N/A	N/A	N/A	N/A	18	30.1	
Cephalosporin	Cefepime	25.3	18	21.3	0	0	N/A	N/A	
	Cefoxitin	N/A	N/A	N/A	N/A	N/A	13.8	30.2	
Monobactams	Aztreonam	N/A	N/A	N/A	0	0	N/A	N/A	
Diaminopyrimidines	Trimethoprim	N/A	N/A	N/A	N/A	N/A	14.3	22.2	
Polypeptide	Colistin	N/A	N/A	N/A	2*	2*	N/A	N/A	

Table S1 *Antibiotic disk diffusion data following the EUCAST guidelines and interpretation using EUCAST breakpoints (2022).*

The antibiotic susceptibility profiles of all bacterial strains used in this study were determined by disk diffusion according to standard EUCAST guidelines (2022, Tab. S1). MIC assays against copper oxide nanoparticles were performed according to ISO 20776-1:2020, with the exception that dilutions were performed with a nine-in-ten dilutions rather than the two-fold dilutions described in the standard to provide greater sensitivity. MBC assays were performed by spotting 10 µl aliquots from each well onto MHA and incubation overnight at 37°C.

Sample	Total Cu (ppb)	Cu (mg L^{-1})
**Water used to create media**	65000 ± 29000	65 ± 29
**Artificial saliva**	65000 ± 29000	65 ± 29
**Leached artificial saliva media**	73000 ± 6000	73 ± 6
**Total leached**	800 ± 35000	8 ± 35

Table S2 Leaching data taken from artificial saliva medium and water using ICP-MS. Copper present in the salts of the medium and water were subtracted from the leached media to assess the trace copper leached from the coated tubing. ICP-MS was repeated in biological triplicate and averaged across ten measurements. Error calculated by averages of thirty total measurements and combining artificial saliva medium plus the leached medium.
